# The Coagulation and Immune Systems Are Directly Linked through the Activation of Interleukin-1α by Thrombin

**DOI:** 10.1016/j.immuni.2019.03.003

**Published:** 2019-04-16

**Authors:** Laura C. Burzynski, Melanie Humphry, Katerina Pyrillou, Kimberley A. Wiggins, Julie N.E. Chan, Nichola Figg, Lauren L. Kitt, Charlotte Summers, Kate C. Tatham, Paul B. Martin, Martin R. Bennett, Murray C.H. Clarke

**Affiliations:** 1 Division of Cardiovascular Medicine, University of Cambridge, Addenbrooke’s Hospital, Cambridge, CB2 0QQ, UK; 2 Division of Anaesthesia, Department of Medicine, University of Cambridge, Addenbrooke’s Hospital, Cambridge, CB2 0QQ, UK; 3 Section of Anaesthetics, Pain Medicine and Intensive Care, Department of Surgery and Cancer, Faculty of Medicine, Imperial College London, London, SW7 2AZ, UK; 4 School of Biochemistry, Biomedical Sciences, University of Bristol, Bristol, BS8 1TD, UK

## Abstract

Ancient organisms have a combined coagulation and immune system, and although links between inflammation and hemostasis exist in mammals, they are indirect and slower to act. Here we investigated direct links between mammalian immune and coagulation systems by examining cytokine proproteins for potential thrombin protease consensus sites. We found that interleukin (IL)-1α is directly activated by thrombin. Thrombin cleaved pro-IL-1α at a site perfectly conserved across disparate species, indicating functional importance. Surface pro-IL-1α on macrophages and activated platelets was cleaved and activated by thrombin, while tissue factor, a potent thrombin activator, colocalized with pro-IL-1α in the epidermis. Mice bearing a mutation in the IL-1α thrombin cleavage site (R114Q) exhibited defects in efficient wound healing and rapid thrombopoiesis after acute platelet loss. Thrombin-cleaved IL-1α was detected in humans during sepsis, pointing to the relevance of this pathway for normal physiology and the pathogenesis of inflammatory and thrombotic diseases.

## Highlights

• Mammalian IL-1α contains a highly conserved thrombin consensus site
• Thrombin cleavage leads to IL-1α activation and shedding from the cell surface
• Thrombin activates IL-1α after epidermal wounding and after acute platelet loss
• Thrombin-cleaved IL-1α is also detected in humans during sepsis

## Graphical Abstract

**Figure:**
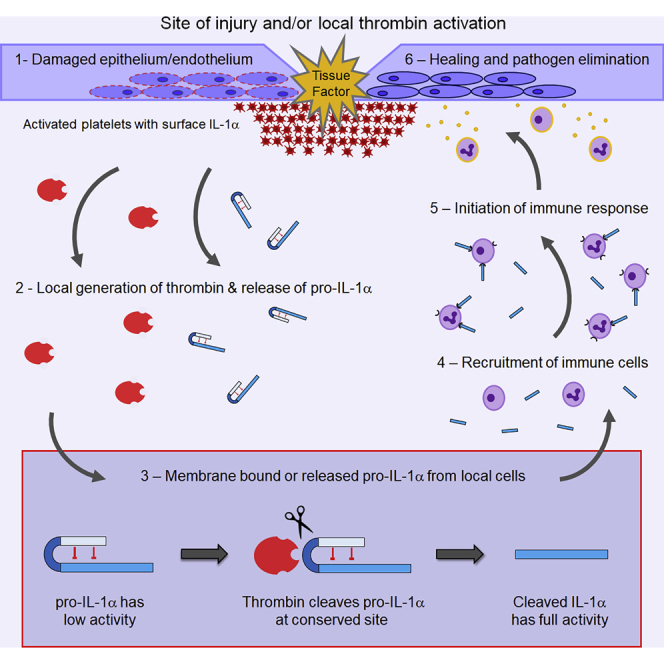

## Teaser

Burzynski et al. reveal that the coagulation protease thrombin directly cleaves pro-interleukin (IL)-1α, rapidly activating the downstream inflammatory cascade. This cleavage site in IL-1α is conserved throughout mammals, suggesting that this link between coagulation and inflammation may be relevant in multiple disease settings.

## Introduction

With the emergence of multicellular life came a greater need to protect against invasion by pathogens and thus the rapid evolution of the immune system. The coagulation system developed from an early innate immune system, with blood serine proteases diverging from complement-like proteases (Delvaeye and Conway, 2009). Bleeding is the primary challenge to survival after wounding, followed by the risk of infection. Thus, activation of inflammation during hemostasis is likely advantageous. Ancient organisms such as horseshoe crabs utilize a combined coagulation and immune system where clotting plugs wounds and entraps pathogens (Delvaeye and Conway, 2009). Although links between coagulation and immunity exist in mammals, they are indirect and slower to act.

Coagulation acts immediately, with the intrinsic or extrinsic pathway activating a protease cascade that drives rapid thrombin activation, fibrin deposition, and platelet activation, leading to hemostasis. Innate immunity is slower and typically requires sensing of pathogen-associated molecular patterns to activate apical cytokines, such as interleukin-1 (IL-1), to direct inflammation and subsequent adaptive immunity (Dinarello, 2009). Inflammation induces tissue factor to promote coagulation, while thrombin induces inflammation via cleavage of protease-activated receptors (PARs) (Delvaeye and Conway, 2009). These slower kinetics could allow microbes to proliferate within, for example, a wound. Hence, a quicker and more direct link between hemostasis and immunity in mammals would benefit host fitness.

IL-1 is ancient with IL-1 homologues identified in echinoderms (Beck and Habicht, 1986). IL-1 signaling via the type 1 IL-1 receptor (IL-1R1) leads to multiple inflammatory effects including vasodilation, increased vascular permeability (Zhu et al., 2012), cytokine secretion, leucocyte recruitment, and upregulation of major histocompatibility complex and co-stimulatory molecules (Dinarello, 2009). IL-1 also effects adaptive immunity by enhancing Th17 differentiation and effector T cell proliferation with Tregs present (Chung et al., 2009, Schenten et al., 2014). These potent effects mean that IL-1 activity is tightly controlled at multiple levels, including a receptor antagonist (IL-1RA), a decoy receptor (IL-1R2), and expression of IL-1α (Zheng et al., 2013, Burzynski et al., 2015) and IL-1β (Black et al., 1988) as proproteins that require proteolysis for full activity. While IL-1β is activated by complex multimeric inflammasomes, IL-1α is cleaved by calpain (Kobayashi et al., 1990) or granzyme B (Afonina et al., 2011). Importantly, increased IL-1 activity is a hallmark of many chronic inflammatory conditions, including rheumatoid arthritis, diabetes, and atherosclerosis.

We identified a direct link between the coagulation and immune systems. IL-1α was activated by thrombin cleavage at a highly conserved site, implying functional importance. We showed key roles for thrombin-cleaved IL-1α in rapid thrombopoiesis after acute platelet loss and for wound healing. We also identified thrombin-cleaved IL-1α in humans with sepsis-associated adult respiratory distress syndrome (ARDS). These findings will likely have widespread implications for inflammatory and thrombotic diseases and normal physiology.

## Results

### Pro-IL-1α Is Cleaved and Activated by Thrombin

**Figure 1:**
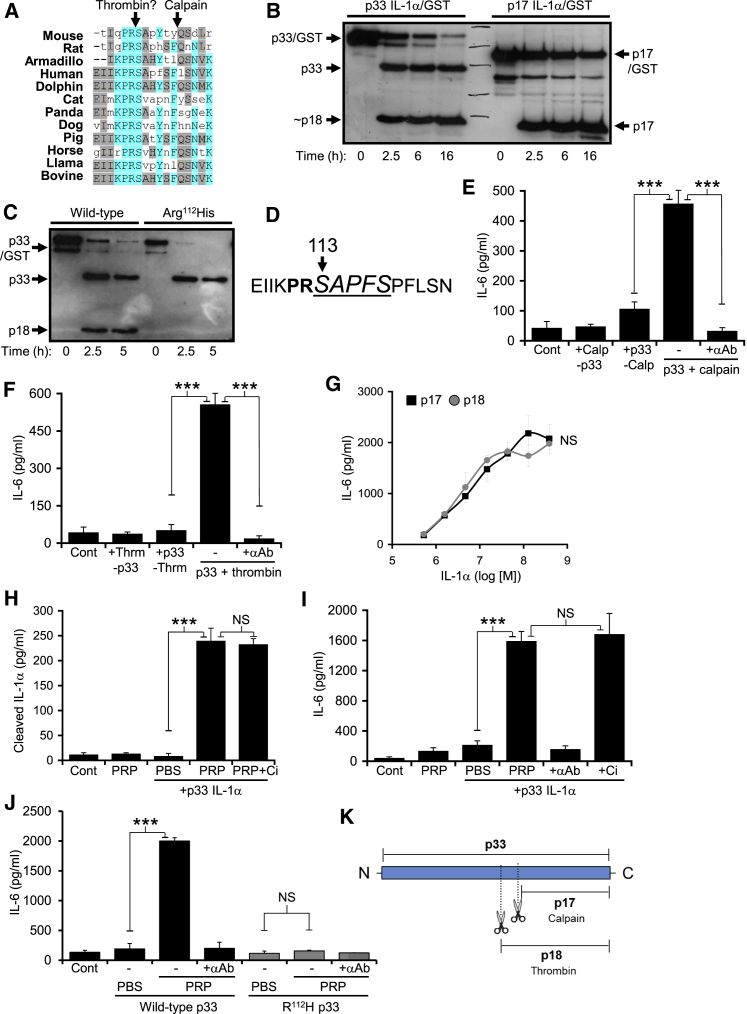
IL-1α Is Activated by Direct Thrombin Cleavage (A) Protein alignment showing conservation of a (K)PRS motif in diverse species. (B and C) Western blots for IL-1α showing cleavage of recombinant p33 to an ∼18kDa form by thrombin (B), and inhibition of cleavage after mutating Arg^112^ to His (C). (D) N-terminal sequencing of thrombin-cleaved human p33 IL-1α detected one sequence (italic underlined) corresponding to processing between Arg^112^ and Ser^113^. (E–G) IL-1-dependent IL-6 production by HeLa cells incubated with calpain cleaved (E) or thrombin cleaved (F) p33 IL-1α, ± a neutralizing IL-1α pAb (+αAb), or increasing concentrations of recombinant p17 or p18 (G). (H and I) Cleavage and activation of p33 IL-1α during clotting of platelet-rich plasma (PRP) as shown with a cleaved IL-1α-specific ELISA (H) or IL-1-dependent IL-6 production by HeLa cells (I), ± a calpain inhibitor (+Ci), or an IL-1α pAb (+αAb). (J) Mutation of Arg^112^ to His prevents p33 activation during clotting of PRP. (K) Pictogram showing cleavage sites within p33 IL-1α. Data represent mean ± SEM; n = 3 (E–G, I), n = 5 (H), n = 2 (J); p = ^∗^≤0.05, ^∗∗^≤0.01, ^∗∗∗^≤0.001; NS = not significant. See also Figure S1.

We investigated direct links between mammalian immune and coagulation systems by examining cytokine proproteins for potential protease consensus sites. IL-1α contained a highly conserved (K)PRS motif, reminiscent of a thrombin consensus, adjacent to the calpain cleavage site (Figure 1A). This PRS site was in 83% of mammalian species with sequences available (Figure S1A), in all orders of mammals except marsupials, and in highly divergent Xenarthrans (e.g., Armadillo). Thrombin treatment of recombinant pro-IL-1α (p33) gave a specific fragment of ∼18kDa (p18), distinct from calpain-matured IL-1α (p17) (Figure 1B). As thrombin typically cleaves after Arg (Gallwitz et al., 2012) we conservatively mutated Arg to His (R^112^H), which prevented cleavage (Figure 1C). Edman degradation confirmed cleavage between Arg^112^ and Ser^113^ (Figure 1D). Cleavage of p33 IL-1α with either calpain (Figure 1E) or thrombin (Figure 1F) fully activated the cytokine, as measured by bioassay with a neutralizing antibody to prove IL-1α activity. Recombinant proteins corresponding to p17 and p18 showed equivalent IL-1 activity (Figure 1G). IL-1R2 could also limit p33 IL-1α activation by thrombin (Figure S1B). p33 IL-1α was cleaved (Figure 1H) and activated (Figure 1I) independent of calpain during ex vivo clotting, while mutant p33 R^112^H was not (Figure 1J), reinforcing the specific action of thrombin. Thus, thrombin specifically cleaved and activated p33 IL-1α at a conserved site adjacent to the calpain site (Figure 1K), revealing a fundamental link between coagulation and immunity.

### Macrophages, Keratinocytes, and Platelets Can Provide Pro-IL-1α for Cleavage by Thrombin

**Figure 2:**
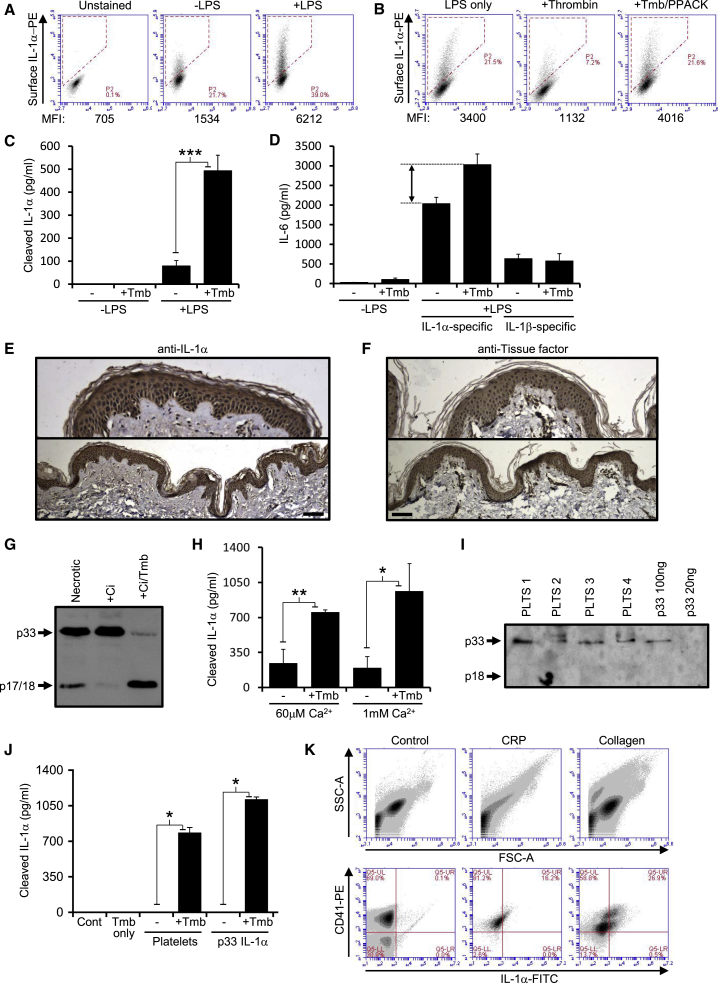
Key Cell Types Contain p33 IL-1α that Can Be Cleaved by Thrombin (A and B) Flow cytometry plots and mean fluorescence intensity (MFI) for cell surface IL-1α in murine J2 macrophages treated ± LPS (A), or LPS followed by thrombin (+Tmb), ± a thrombin inhibitor (PPACK) (B). (C and D) Cleaved IL-1α-specific ELISA (C) and IL-1 activity assay (D) showing release of active p18 from the surface of LPS-treated J2 macrophages into the conditioned media by thrombin. (E and F) Human skin sections stained (brown) for IL-1α (E) or tissue factor (F). (G) Western blot for IL-1α in human keratinocyte necrotic lysates, ± a calpain inhibitor (+Ci), ± thrombin. (H) Cleaved IL-1α-specific ELISA showing thrombin cleavage of p33 IL-1α within human keratinocyte conditioned media. (I) Western blot for IL-1α showing p33 within human platelets. (J) Cleaved IL-1α-specific ELISA showing thrombin cleavage of p33 IL-1α in human platelet lysates or recombinant p33. (K) Flow cytometry for CD41 and IL-1α on resting, and collagen or collagen-related peptide (CRP) treated human platelets. Data represent mean ± SEM; n = 3 (C, D, J), n = 2 (H), n = 5 (K); p = ^∗^≤0.05, ^∗∗^≤0.01, ^∗∗∗^≤0.001. Scale bars represent 100μm. See also Figure S1.

We investigated which cell types could provide p33 IL-1α for activation by thrombin. Macrophages and dendritic cells express p33 on their surface (Kurt-Jones et al., 1985, Fettelschoss et al., 2011), and LPS-treated human and mouse primary macrophages and cell lines increased surface expression of IL-1α (Figures 2A, S1C, and S1D), which could be cleaved by thrombin (Figure 2B) to release active p18 into the conditioned media (Figures 2C and 2D). Epidermal keratinocytes provide barrier function that stops infection, and constitutively express IL-1α (Anttila et al., 1990). Human and mouse skin showed IL-1α expression specifically within the epidermis that colocalized with tissue factor (Figures 2E, 2F, S1E, and S1F), a potent activator of thrombin. Keratinocyte lysates contained p33 IL-1α that was partially processed by calpain, but extensively processed by thrombin (Figure 2G). Keratinocytes also secreted p33 that could be processed by thrombin (Figure 2H), suggesting thrombin cleavage of epidermal IL-1α after wounding could be an elegant mechanism to rapidly alert the immune system and safeguard against infection. Platelets are in close proximity to thrombin during hemostasis, and contain IL-1α and IL-1β (Hawrylowicz et al., 1989). Resting platelets contained p33 IL-1α (Figure 2I), while platelet lysates contained p33 that could be cleaved by thrombin (Figure 2J). Platelet activation with collagen or collagen-related peptide (CRP) resulted in microvesiculation and surface IL-1α expression on human (Figure 2K) and mouse (Figure S1G) platelets. Similarly, platelet activation increased the amount of p33 IL-1α that thrombin could release from the surface (Figure S1H). Thus, key cell types at sites of coagulation release and/or present IL-1α on their surface, which can be cleaved and activated by thrombin.

### Generation of a Mouse Model in Which IL-1α Cannot Be Activated by Thrombin

**Figure 3:**
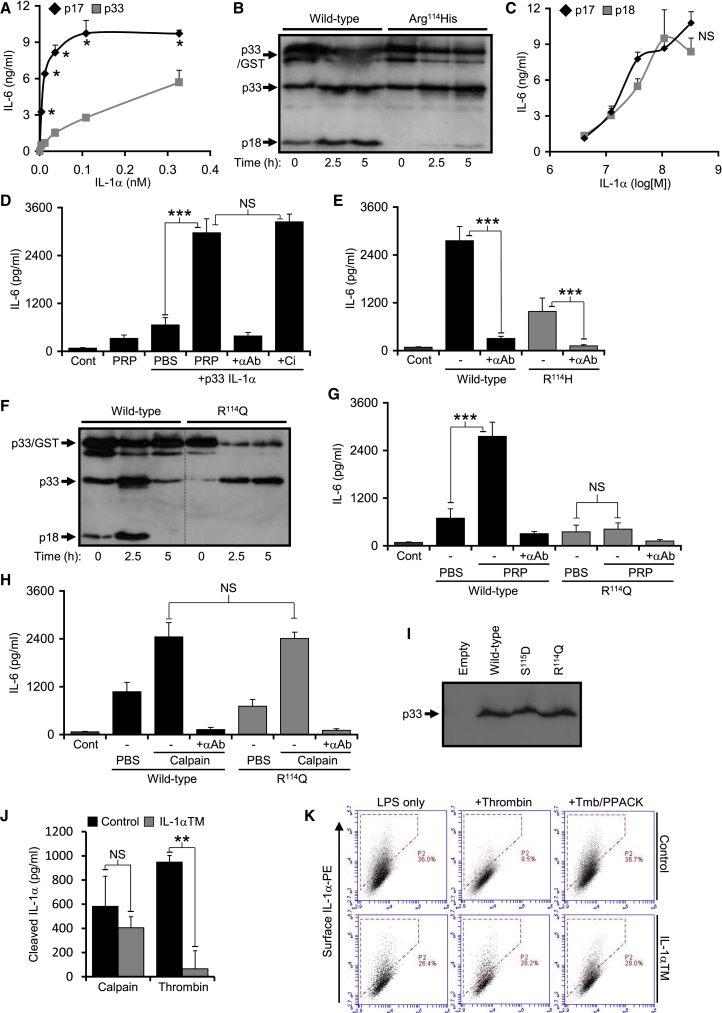
Generation of a Mouse Model in which IL-1α Cannot Be Activated by Thrombin (A) IL-1-dependent IL-6 production by murine fibroblasts incubated with increasing concentrations of recombinant mouse p17 or p33 IL-1α. (B) Western blot for IL-1α showing thrombin cleavage of recombinant mouse wild-type p33 to a ∼18kDa form, but less cleavage of Arg^114^His p33. (C–E) IL-1-dependent IL-6 production by murine fibroblasts incubated with increasing concentrations of recombinant mouse p17 or p18 (C), or wild-type (D) and Arg^114^His mutant (E) p33 incubated with clotting platelet-rich plasma (PRP), ± a calpain inhibitor (+Ci) and/or an IL-1α pAb (+αAb). (F) Western blot for IL-1α showing no thrombin cleavage of an Arg^114^Gln mutant p33. (G and H) IL-1-dependent IL-6 production by murine fibroblasts incubated with wild-type or mutant p33 incubated with clotting platelet-rich plasma (G) or active calpain (H). (I) Western blot for IL-1α showing equivalent expression of exogenous wild-type or mutant p33 in HeLa cells. (J) Cleaved IL-1α-specific ELISA reporting the level of calpain or thrombin processing of p33 IL-1α derived from LPS-treated control or IL-1α thrombin mutant (IL-1α TM) mBMDMs. (K) Flow cytometry for surface IL-1α on LPS-treated control or IL-1α TM mBMDMs incubated ±thrombin (+Tmb) and ± a thrombin inhibitor (PPACK). Data represent mean ± SEM; n = 3 (A, E, and J), n = 2 (C and H), n = 5 (G); p = ^∗^≤0.05, ^∗∗^≤0.01, ^∗∗∗^≤0.001; NS = not significant. See also Figures S2 and S3, and Table S1.

To establish in vivo relevance of p18 IL-1α we developed a mouse model in which IL-1α cannot be activated by thrombin. Whether mouse IL-1α requires cleavage for full activity has not been shown. Concentration-response curves (Figure 3A) and cleavage of p33 (Figure S2A) showed that mouse IL-1α also required cleavage for full activity. Mouse p33 was also cleaved (Figure 3B) and activated (Figure S2B) by thrombin, with mutation of the equivalent site (R^114^H) reducing cleavage (Figure 3B). Again, recombinant mouse p17 and p18 showed equivalent activity (Figure 3C), and mouse p33 was also activated independently of calpain during ex vivo clotting (Figure 3D). However, the remnant thrombin cleavage of mouse R^114^H p33 still produced active IL-1α (Figure 3E). We tested a panel of other mutations for thrombin cleavage by western (Figure S2C) and ELISA (Figure S2D), and relative increase in activity during ex-vivo clotting (Figure S2E). The R^114^Q mutant was resistant to thrombin cleavage in vitro (Figure 3F) and showed no increase in activity after clotting (Figure 3G). R^114^Q was cleaved by calpain (Figure 3H) and other proteases (Figure S2F) equivalently to wild type, was expressed at the same protein level (Figure 3I), and maintained comparable low basal activity (Figure S2G). The R^114^Q point mutation was generated in the murine *Il1a* locus and offspring bred to homozygosity to generate IL-1α thrombin mutant mice (IL-1α TM). Testing macrophage-derived p33 IL-1α revealed equivalent calpain cleavage in both genotypes, but an inability of thrombin to cleave p33 from IL-1α TM macrophages (Figure 3J). Thrombin could not cleave surface IL-1α from IL-1α TM macrophages, (Figure 3K). No phenotypic difference was found between control and IL-1α TM mice with regard to: full blood count or clotting parameters (Table S1 and Figure S2H); IL-1α and IL-1β expression, cell surface IL-1α, IL-1α cleavage upon necrosis or IL-1α and IL-1β release after inflammasome activation (Figure S3A–S3D); Ly6C^Hi^ monocytes (Figure S3E); spleen or lymph node weight (Figure S3F and S3G); splenic CD4 and CD8 T cell or Treg frequency (Figure S3H and S3I); or splenic CD4 and CD8 T cell activation (Figure S3J and S3K). This data proves that the mutation functions as shown in vitro and did not created any general phenotypic differences.

### p18 IL-1α Drives Rapid Thrombopoiesis and Wound Healing, and Is Generated during Sepsis in Humans

**Figure 4:**
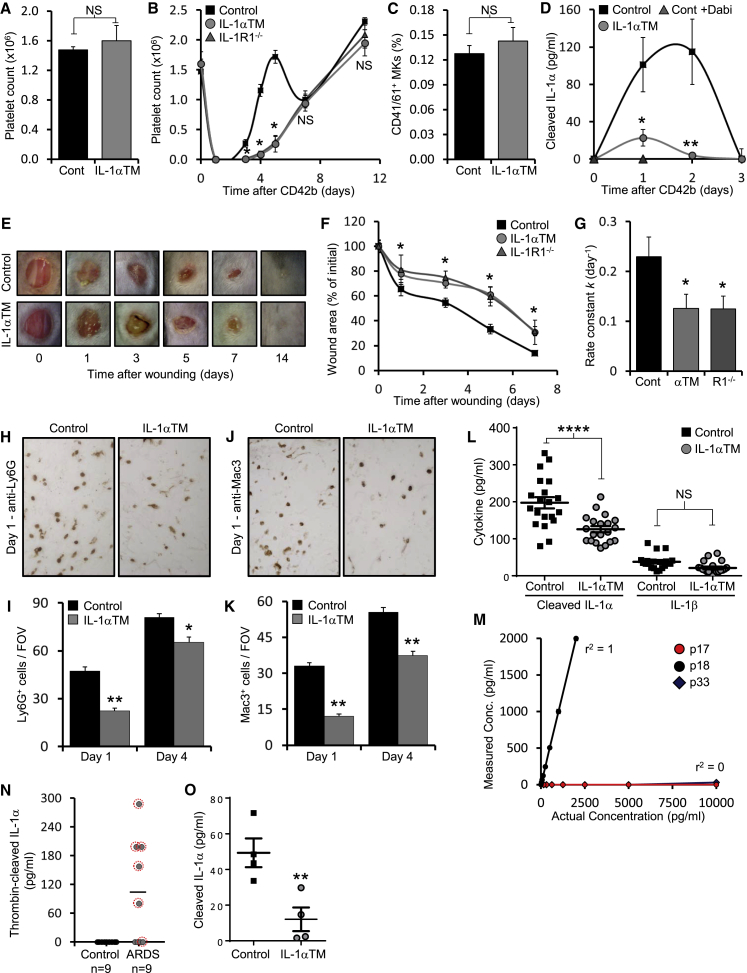
p18 IL-1α Drives Rapid Thrombopoiesis and Wound Healing, and Is Generated during Sepsis in Humans (A and B) Platelet count by flow cytometry in mice under basal conditions (A), or over time after anti-CD42b-mediated platelet depletion (B). (C) Flow cytometry for the number of CD41^+^ and CD61^+^ MKs in the bone marrow of control and IL-1α TM mice under basal conditions. (D) Cleaved IL-1α-specific ELISA reporting the level of cleaved IL-1α within the serum of control mice treated ±dabigatran (+Dabi) and IL-1α TM mice over time after anti-CD42b-mediated platelet depletion. (E–G) Representative images showing gross healing of 4 mm excisional skin wounds (E), quantitation of wound area (F) over time, and rate of closure (G) in mice as indicated. (H–K) Representative images and quantitation of Ly6G+ neutrophils (H and I) or Mac3+ macrophages (J and K) recruited to the granulation tissue underlying wounds at the times indicated. (L) ELISA data showing release of cleaved IL-1α or IL-1β from wounded skin. (M) Sandwich ELISA data showing reactivity of the p18-specific ELISA to p17, p18, or p33 IL-1α. (N and O) p18-specific ELISA data reporting level of p18 in plasma from control individuals or patients with sepsis-associated ARDS (N). Red circles indicate +VE microbiology in bronchoalveolar lavage fluid. (O) Cleaved IL-1α-specific ELISA reporting the level of cleaved IL-1α within the serum of control and IL-1α TM mice during LPS-induced endotoxemia. Data represent mean ± SEM; n = ≥4 (A, B, and D), n = 3 (C), n = ≥5 (F and G), n = ≥10 wounds (I and K), n = 20 wounds (L), n = 2 (M); p = ^∗^≤0.05, ^∗∗^≤0.01, ^∗∗∗^≤0.001; NS = not significant. See also Figure S4 and Table S2, S3.

Conservation of the thrombin cleavage site implies positive selection and thus an advantage on fitness. IL-1α drives rapid thrombopoiesis after acute platelet loss (Nishimura et al., 2015), but how IL-1α is activated is unknown. We postulated that thrombin could generate active IL-1α after platelet loss or consumption during coagulation. We treated control and IL-1α TM mice with anti-CD42b to deplete platelets and measured platelet rebound over time. Platelet count between groups was the same before depletion (Figure 4A). In control mice platelet count was reinstated within 4–5 days (Figure 4B), followed by a drop consistent with normal platelet turnover after IL-1α-dependent fragmentation of all mature megakaryocytes (MKs) (Nishimura et al., 2015). IL-1α TM mice showed delayed platelet recovery with counts only returning to baseline after 10–11 days (Figure 4B)—kinetics consistent with thrombopoietin (TPO)-driven MK differentiation (Machlus and Italiano, 2013) and near identical to platelet rebound in *Il1r1*^-/-^ mice (Figure 4B). Bone marrow MK number was similar between groups before platelet depletion (Figure 4C) and repeated blood sampling had minimal effect on platelet count in non-depleted mice (Figure S4A). The transient increase in cleaved IL-1α in serum after platelet depletion (Nishimura et al., 2015) was near absent in IL-1α TM mice, and in control mice after thrombin inhibition (Figure 4D), supporting failure of thrombin to generate p18 IL-1α. Platelet depletion caused multiple hemorrhages (Figure S4B), which could activate prothrombin via tissue factor. This suggests thrombin activation of IL-1α is the mechanism that induces rapid platelet production after acute platelet consumption.

Given the colocalization of IL-1α with tissue factor in the epidermis (Figures 2E and 2F) we examined whether failure to generate p18 in IL-1α TM mice affects wound healing—a process combining initial hemostasis and subsequent immune cell recruitment to direct wound repair (Eming et al., 2014). IL-1α TM mice showed significantly delayed excisional wound closure at all stages of repair (Figures 4E and 4F), with a rate of wound closure ∼1.8-fold slower than control mice (Figure 4G), which was equivalent to wound repair in *Il1r1*^-/-^ mice (Figures 4F and 4G). Fewer neutrophils (Figures 4H and 4I) and monocyte/macrophages (Figures 4J and 4K) were recruited to the granulation tissue underlying IL-1α TM wounds, and less cleaved IL-1α was released by wounded IL-1α TM skin (Figure 4L), with both groups releasing equivalent IL-1β, and active thrombin (Figure S4C). Thrombin inhibition reduced cleaved IL-1α production from wounds in control mice (Figure S4D), although scarring was similar between groups (Figure S4E). This suggests that thrombin cleaved IL-1α is produced after epidermal injury and that it drives immune cell recruitment and healing.

To examine whether p18 IL-1α is produced in humans in vivo, we generated a peptide antibody specific to p18 and p33, but not p17 (Figure S4F and S4G) and developed this into a sandwich ELISA specific for p18 IL-1α (Figure 4M). Generalized sepsis activates coagulation in humans (Delvaeye and Conway, 2009), with sepsis-associated ARDS showing more severe coagulation abnormalities (Abraham, 2000). Examination of sera from ARDS and age-matched control individuals (Table S2) revealed no p18 in control individuals, but p18 generation in sepsis-associated ARDS (Figure 4N), with all patient sera that contained p18 IL-1α also positive for microbiology in bronchoalveolar lavage fluid (Figure 4N and Table S3). Lastly, cleaved IL-1α level was lower during experimental endotoxemia in IL-1α TM mice (Figure 4O), implying generation of p18 IL-1α during human sepsis and mouse endotoxemia. Outcome of endotoxemia was unaffected in IL-1α TM mice (Figure S4H), consistent with previous findings in *Il1r1*^-/-^ mice (Kayagaki et al., 2011). Thus, thrombin activated IL-1α drives two physiological processes in vivo in mice and is generated in humans during generalized infection.

## Discussion

Selection pressure drives biological complexity, with co-evolution of hosts and pathogens creating ever more sophisticated defense mechanisms. Primitive genes underwent subfunctionalization to generate the coagulation, immune, and complement systems we see in mammals today. Crosstalk between these systems is thought to have evolved to increase host fitness, but this interplay may represent conserved pathways from the combined ancestral coagulation-immune system.

Here we revealed a direct link between the coagulation and immune systems. IL-1α was activated by thrombin at a conserved site, implying functional importance. Macrophages, keratinocytes, and platelets expressed p33 IL-1α that was activated and released by thrombin. Experiments in a mouse model in which IL-1α cannot be activated by thrombin revealed roles for p18 IL-1α in rapid thrombopoiesis after acute platelet loss and wound healing. Importantly, we also identified p18 IL-1α in humans with sepsis-associated ARDS.

pro-IL-1α contains 271 amino acids, with all cytokine activity in the C-terminus from residue 120 onwards. Conserved regions form the distinctive beta trefoil fold in the C-terminus, and a nuclear localization sequence in the N-terminus (Rivers-Auty et al., 2018). Thus, the perfect conservation of the IL-1α PRS sequence in 83% of mammalian species is likely due to it forming a thrombin site under positive selection. All orders of mammal, except marsupials, showed the PRS site, with the distant Xenarthrans (e.g. Armadillo) having undergone 240 million years of divergent evolution from primates (Nishihara et al., 2009). Before the emergence of IL-1α and IL-1β, all ancestral IL-1 sequences are annotated as IL-1β (e.g. birds, reptiles). However, some species contained PRS sequences and others showed high conservation of PVRS adjacent to the caspase-1 site in ancestral IL-1, and thus in a position analogous to the IL-1α PRS site. This suggests the potential divergence of an ancestral IL-1 cleaved by both caspases and thrombin, to IL-1β cleaved by caspases and IL-1α cleaved by thrombin and calpain. Coagulation and immunity are combined in most arthropods (Delvaeye and Conway, 2009), with wounding perhaps the most ancient danger signal. In Drosophila, the Toll signaling pathway utilizes myeloid differentiation primary response 88 (MyD88) and Toll/IL-1 receptor (TIR) signaling adaptors homologous to those used by mammalian IL-1R1. Intriguingly, cleavage of the Toll ligand Spätzle to a form that induces TIR-MyD88 signaling can be via the thrombin-like *gastrulation defective, snake*, *and easter* (Hecht and Anderson, 1992, Kimbrell and Beutler, 2001). This suggests that an ancient proteolytic cascade in an insect that diverged ∼600 million years ago still has a functional equivalent in mammals today.

Conservation of the IL-1α thrombin site implies positive selection, and thus a fitness advantage. The simplest reason to instigate an immune response during coagulation is to prevent wound infection. As human and mouse epidermis showed colocalization of IL-1α with tissue factor, any laceration damaging cutaneous vessels would bring together p33 and active thrombin. Indeed, slower wound healing in IL-1α TM mice, indicating p18 IL-1α was required, matches the retarded healing in *Il1r1*^-/-^ mice (Lee et al., 2017). It will be interesting to see whether other interfaces between host and the external environment show similar expression of IL-1α and tissue factor, e.g., mucosal epithelium. The current principal link between coagulation and immunity is cleavage of PARs by thrombin, which produces cytokines and inflammation. Cytokines can take ∼6 h before peak production, providing time for bacterial growth. However, IL-1 drives rapid vasodilation (Wakabayashi et al., 1991), and increases vascular permeability via a *MYD88*-*ARNO*-*ARF6* pathway within 15 mins (Zhu et al., 2012), enabling rapid extravasation of complement and leucocytes to sites of injury and/or infection. Thus, we envisage p33 cleavage from the surface of macrophages or platelets by thrombin to represent a rapid IL-1-mediated defense mechanism.

Another key finding is that p18 IL-1α drove rapid platelet production after acute platelet loss. MKs can utilize caspases to produce platelets (Clarke et al., 2003), and IL-1α induces caspase-3-dependent MK fragmentation to rapidly release platelets in a TPO-independent manner (Nishimura et al., 2015). After acute platelet loss, this alternative pathway quickly generates functional platelets to enable hemostasis, with TPO maintaining steady-state platelet count (Nishimura et al., 2015). How IL-1α is activated in these models was unknown. We showed delayed platelet recovery after depletion in IL-1αTM mice, which only differ from controls by a single amino acid change that blocked thrombin cleavage of IL-1α, suggesting p18 is generated by thrombin after platelet loss. We propose two mechanisms for thrombin activation: firstly, thrombin is activated when platelets are “consumed” during hemostasis, and thrombin cleaved IL-1α from the platelet surface; secondly, thrombocytopenia causes microvessel leakage (purpura, petechiae) where prothrombin is exposed to and activated by tissue factor. Although purpura and petechiae are seen in skin, microvessel leakage occurs systemically and thus any local source of p33 IL-1α could be activated. In both cases, p18 IL-1α level would inversely correlate to platelet count. Thus, platelet loss by any cause would lead to thrombin activation and potential cleavage of local p33 IL-1α to induce rapid platelet shedding from MKs. IL-1 also enables rapid myeloid recovery after acute bone-marrow injury (Pietras et al., 2016), suggesting that IL-1 can promote other emergency programs after acute insults.

The identification of a direct link between the coagulation system and the activation of the IL-1α inflammatory cascade raises important questions. The current findings are limited to wound healing and acute platelet loss settings, and thus it will be necessary to determine the relevance of p18 IL-1α in pathological settings where one might expect this pathway to be important. For example, although we showed p18 IL-1α is generated during sepsis-associated ARDS, it would be valuable to determine whether p18 affects or correlates (e.g. as a biomarker) to clinical outcome. The day-to-day heterogeneity of patients and clinical interventions within these extreme phenotypes would require a prospective study and modelling (e.g., a Cox’s proportional-hazard model) to identify any effect p18 contributed. Many diseases are driven by the interplay between coagulation and inflammation. Inflammation drives atherosclerosis and IL-1α can play a dominant role independent of inflammasomes (Menu et al., 2011, Freigang et al., 2013), suggesting another mechanism activates IL-1α. Plaques contain thrombin-antithrombin complexes (Borissoff et al., 2010) and show fibrin localized throughout (Smith, 1986), implying thrombin activation occurs throughout atherogenesis. Thus, p18 IL-1α might drive atherogenesis, which could be tested using fat-fed IL-1α TM/*Apoe*^-/-^ mice. Patients with aneurysm have IL-1α levels directly correlated to the size of the overlying mural thrombus, and levels fall after repair (Yates et al., 2011), suggesting that thrombus may produce IL-1α in vivo. Indeed, p18 could be produced and subsequently trapped in developing thrombi, with potential release during fibrinolysis recruiting leukocytes to help with clot resolution. These potential effects of p18 IL-1α on thrombi could be studied with intravital microscopy. Platelet IL-1α also drives arthritis (Boilard et al., 2010) and cerebrovascular inflammation after ischaemia (Thornton et al., 2010), and thus platelet-derived p18 IL-1α may be involved in these settings too. Further studies should shed light on the relevance of thrombin-mediated IL-1α activation in these settings.

## STAR★Methods

### Key Resources Table

**Table:** 

REAGENT or RESOURCE	SOURCE	IDENTIFIER
**Antibodies**		

Rabbit polyclonal anti-human IL-1α	Peprotech	Cat#500-P21A; RRID:AB_147894
Goat polyclonal anti-human IL-1α	R&D	Cat#AF-200-NA; RRID:AB_354386
Goat polyclonal anti-human IL-1α	Abcam	Cat#ab7632; RRID:AB_306001
Rabbit polyclonal anti-human p18 IL-1α	Innovagen	N/A
Rabbit polyclonal anti-human biotinylated p18 IL-1α	This paper	N/A
Goat polyclonal anti-mouse IL-1α	R&D	Cat#AF-400-NA; RRID:AB_354473
Goat polyclonal anti-human IL-1β	R&D	Cat#AF-201-NA; RRID:AB_354387
Mouse monoclonal anti-human IL-1β (Clone 8516)	R&D	Cat#MAB201; RRID:AB_358006
Rabbit polyclonal anti-mouse IL-1β	Peprotech	Cat#500-P51; RRID:AB_147630
Mouse monoclonal anti-human IL-1α-FITC (Clone 3405)	R&D	Cat#FAB200F; RRID:AB_357119
Hamster monoclonal anti-mouse IL-1α-PE (Clone ALF-161)	Biolegend	Cat#503203; RRID: AB_315281
Rat monoclonal anti-mouse CD42b	Emfret	Cat#R300; RRID:AB_2721041
Rat monoclonal anti-mouse CD41-FITC (Clone MWReg30)	Biolegend	Cat# 133904; RRID: AB_2129746
Hamster monoclonal anti-mouse/rat CD61-PE (Clone 2C9.G2)	Biolegend	Cat#104308; RRID: AB_313085
Rat monoclonal anti-mouse CD4-FITC (Clone RM4-5)	eBioscience	Cat#11-0042-82; RRID: AB_464896
Rat monoclonal anti-mouse CD8-PerCP (Clone 53-6.7)	Biolegend	Cat#100732; RRID: AB_893423
Rat monoclonal anti-mouse CD62L-PE (Clone MEL-14)	Biolegend	Cat#104408; RRID: AB_313095
Rat monoclonal anti-mouse CD44-APC (Clone IM7)	Biolegend	Cat#103012; RRID: AB_312963
Rat monoclonal anti-mouse CD25-APC (Clone 3C7)	Biolegend	Cat#101909; RRID: AB_961208
Rat monoclonal anti-mouse FOXP3-PE (Clone MF-14)	Biolegend	Cat#126403; RRID: AB_1089118
Rat monoclonal anti-mouse IL-10-APC (Clone JES5-6E3)	Biolegend	Cat#505010; RRID: AB_315364
Rat monoclonal anti-mouse IL-17-PE (Clone TC11-18H10.1)	Biolegend	Cat#506904; RRID: AB_315464
Rat monoclonal anti-mouse IFNγ-FITC (Clone XMG1.2)	Biolegend	Cat#505806; RRID: AB_315400
Rat monoclonal anti-mouse IFNγ-FITC (Clone XMG1.2)	Biolegend	Cat#505826; RRID: AB_2295770
Rat monoclonal anti-mouse CD11b-Alexa Fluor 488 (Clone M1/70)	Biolegend	Cat#101217; RRID: AB_389305
Rat monoclonal anti-mouse CD115-PE (Clone AFS98)	eBioscience	Cat#12-1152-82; RRID:AB_465808
Rat monoclonal anti-mouse Ly6G-PE/Cy7 (Clone 1A8)	Biolegend	Cat#127618; RRID: AB_1877261
Rat monoclonal anti-mouse Ly6C- Alexa Fluor 647 (Clone 7/4)	AbD Serotec	Cat#MCA771GA; RRID:AB_324243
Mouse monoclonal anti-human CD41-PE (Clone HIP8)	Biolegend	Cat#303706; RRID: AB_314376
Rat monoclonal anti-mouse CD41-FITC (Clone MWReg30)	Biolegend	Cat#133904; RRID: AB_2129746
Rabbit polyclonal anti-human α/β-Tubulin	Cell Signaling Technology	Cat#2148S; RRID: AB_2288042
Rabbit monoclonal anti-human Tissue Factor (Clone EPR8986)	Abcam	Cat#ab151748
Rat monoclonal anti-mouse Ly6G (Clone 1A8)	Biolegend	Cat#127602; RRID: AB_1089180
Rat monoclonal anti-mouse Mac3 (Clone M3/84)	BD	Cat# 553322; RRID:AB_394780
Polyclonal Goat Anti-Rabbit Biotinylated Immunoglobulins	Dako	Cat#E0432; RRID:AB_2313609
Goat anti-rabbit IgG HRP	GE	Cat#NA934; RRID:AB_772206
Bovine anti-goat IgG HRP	Jackson	Cat#805-035-180; RRID:AB_2340874
Anti-rat IgG	Vector	Cat#I-4000; RRID:AB_2336356
Anti-rabbit IgG	Vector	Cat#I-1000; RRID:AB_2336355
Goat anti-rabbit IgG HRP	Dako	Cat#P0448; RRID:AB_2617138
TruStain FcX	Biolegend	Cat#101320, AB_1574975

**Bacterial and Virus Strains**		

pGEX-4T-3	GE	Cat#27-4583-01
Rosetta2 (DE3) pLysS	Novagen	Cat#71403
XL10 Gold	Agilent Technologies	Cat#200314

**Biological Samples**		

Paraffin embedded human skin sections	Abcam	Cat#ab4365
Paraffin embedded mouse skin sections	This paper	N/A
Sepsis-associated ASDS clinical samples	Collected under research ethics: 08/H0306/17 – *‘Fate and Function of neutrophils in acute lung injury’*	N/A

**Chemicals, Peptides, and Recombinant Proteins**		

Recombinant Human IL-1α	Peprotech	Cat#200-01A
Recombinant Mouse IL-1β	Peprotech	Cat#211-11B
Recombinant Human p17 IL-1α	This paper	N/A
Recombinant Human p18 IL-1α	This paper	N/A
Recombinant Human p33 IL-1α	This paper	N/A
Recombinant Mouse p17 IL-1α	This paper	N/A
Recombinant Mouse p18 IL-1α	This paper	N/A
Recombinant Mouse p33 IL-1α	This paper	N/A
Recombinant Human IL-1 R2	R&D	Cat#663-2R
Recombinant Human IL-1RAcP	Sino	Cat#10121-H08H
p18 Immunizing peptide	Innovagen	N/A
Calpeptin	Enzo	Cat# BML-PI101-0010
LPS	Invivogen	Cat#tlrl-3pelps
LPS	Sigma	Cat#L2630
Nigericin	Calbiochem	Cat#481990
Dabigatran etexilate	Pradaxa	N/A
RBC Lysis Buffer	eBioscience	Cat#00-4300-54
FOXP3 Fix/Perm Buffer Set	Biolegend	Cat#421403
Cell Activation Cocktail (ionomycin/PMA/brefeldin A)	Biolegend	Cat#423303
Intracellular Staining Permeabilization Wash Buffer	Biolegend	Cat#421002
Collagen related peptide	Professor Richard Farndale, Dept. Biochemistry, Cambridge.	https://collagentoolkit.bio.cam.ac.uk/thp/generic
Collagen	Helena	Cat#5368
Restriction grade thrombin	Merck	Cat#69671
Chymase	Sigma	Cat#C8118
Elastase	Enzo	Cat#BML-SE284-0100
Granzyme B	Biovision	Cat#7233-10
PPACK	Enzo	Cat#BML-PI117
RIPA Lysis and Extraction Buffer	ThermoFisher	Cat#89900
ECL Western Blotting Detection Reagent	Amersham	Cat#RPN2106
Streptavidin-PE	R&D	Cat#12-4317-87
TMB Substrate Kit	ThermoFisher	Cat#34021
Vectastain ABC HRP kit	Vector	Cat#PK-6100
DAB Peroxide substrate	Vector	Cat#SK-4105
Envision detection kit	Dako	Cat#K500711-2
Mouse and Rabbit Specific HRP/DAB (ABC) Detection kit	Abcam	Cat#ab64264
Picrosirius Red Stain Kit	Polysciences	Cat#24901

**Critical Commercial Assays**		

Human IL-1α ELISA Development Kit	Peprotech	Cat#900-T11
Murine IL-1α ELISA Development Kit	Peprotech	Cat#900-K82
IL-1α Mouse ProcartaPlex Simplex Kit	ThermoFisher	Cat#EPX01A-20611-901; RRID: AB_2575923
IL-1β Mouse ProcartaPlex Simplex Kit	ThermoFisher	Cat#EPX01A-26002-901; RRID: AB_2575930
IL-6 Mouse Flex set	BD	Cat#558301
IL-1α Human ProcartaPlex Simplex Kit	ThermoFisher	Cat#EPX01A-10243-901; RRID: AB_2575794
IL-1β Human ProcartaPlex Simplex Kit	ThermoFisher	Cat#EPX01A-10224-901; RRID: AB_2575782
IL-6 Human Flex set	BD	Cat#558276
Mouse Thrombin-Antithrombin Complexes ELISA Kit	Abcam	Cat#ab137994
V-PLEX human biomarker 40-plex kit	Mesoscale Discovery	K15209D

**Experimental Models: Cell Lines**		

Human: HeLa cell line	ECACC	Cat#93021013; RRID: CVCL_0030
Mouse: adult fibroblast cells	This paper	N/A
Mouse: bone marrow derived fibroblast cells	This paper	N/A
Human: U937 cell line	ECACC	Cat#85011440; RRID:CVCL_0007
Mouse: J2 cell line	Mouse bone marrow-derived macrophages immortalized with a J2 virus.	See PMID: 18604214
Human: monocyte-derived macrophage cells	This paper	N/A
Human: keratinocyte cells	Gibco	Cat#A13401

**Experimental Models: Organisms/Strains**		

Mouse: IL-1αTM	inGenious Targeting Laboratory	N/A
Mouse: IL-1R1-/-, B6.129S7-Il1r1tm1Imx/J	The Jackson Laboratory	Cat#003245; RRID:IMSR_JAX:003245

**Oligonucleotides**		

Primer: human IL1α R112H Forward: GAAATCATCAAGCCTCACTCAGCACCTTTTAG	This paper	N/A
Primer: human IL1α R112H Reverse: CTAAAAGGTGCTGAGTGAGGCTTGATGATTTC	This paper	N/A
Primer: mouse IL1α R114Q Forward: GAGACCATCCAACCCCAATCAGCACCTTACACC	This paper	N/A
Primer: mouse IL1α R114Q Reverse: GGTGTAAGGTGCTGATTGGGGTTGGATGGTCTC	This paper	N/A
Primer: mouse IL1α R114H Forward: GAGACCATCCAACCCCACTCAGCACCTTACACC	This paper	N/A
Primer: mouse IL1α R114H Reverse: GGTGTAAGGTGCTGAGTGGGGTTGGATGGTCTC	This paper	N/A
Primer: mouse IL1α S115D Forward: ACCATCCAACCCAGAGACGCACCTTACACCTAC	This paper	N/A
Primer: mouse IL1α S115D Reverse: GTAGGTGTAAGGTGCGTCTCTGGGTTGGATGGT	This paper	N/A

**Software and Algorithms**		

Graphpad Prism 6	Graphpad Software	https://www.graphpad.com/scientific-software/prism/
FlowCytomix Pro 3.0	eBiosciences	Discontinued
Image-Pro Insight 9.1	Media Cybernetics	http://www.mediacy.com/imagepro
ImageJ 1.5	National Institutes of Health	https://imagej.nih.gov/ij/index.html
ImageJ FibrilTool	Boudaoud et al., 2014 PMID: 24481272	https://www.nature.com/articles/nprot.2014.024#procedure

**Other**		

Biopsy punch	Kai Medical	Cat#BP-40F
GSTrap HP column	GE	Cat#17-5281-01
EpiLife medium	ThermoFisher	Cat#MEPI500CA
CL-XPosure Film	ThermoFisher	Cat#34089

### Contact for Reagent and Resource Sharing

Further information and requests for resources and reagents should be directed to and will be fulfilled by the Lead Contact, Murray Clarke (mchc2@cam.ac.uk).

### Experimental Model and Subject Details

#### Cell culture

HeLa or mouse fibroblasts were cultured in DMEM, 10% FCS, 100U/ml penicillin, 0.1mg/ml streptomycin, 2mM L-glutamine. U937, J2 immortalised macrophages and mouse BMDMs were cultured in RPMI 1640, 10% FCS, 2mM L-glutamine, 100U/ml penicillin, 0.1mg/ml streptomycin, and 50μM β-mercaptoethanol, with 15% L929 conditioned media for mBMDM differentiation. Mouse fibroblasts and BMDMs cultures were derived from adult male and female mice. For human monocyte-derived macrophages (hMDMs), monocytes were isolated from whole blood (male and female adults) following citration, dextran sedimentation, and plasma-percoll density gradient centrifugation. hMDMs were differentiated by culturing adherent monocytes (5–7 d) Iscove's DMEM with 100U/ml penicillin, 0.1mg/ml streptomycin, 2mM L-glutamine and 10% autologous serum, with washing and media replacement every ∼2 days. Keratinocytes were cultured in EpiLife media (Thermo Fisher) with Ca^2+^ added as indicated. All cells were grown at 37°C using standard cell culture techniques and were frequently tested for mycoplasma infection. Cell lines were recently obtained commercially (e.g. HeLa and U937) and authenticated by the company (ECACC). Primary cells were differentiated in house (e.g. mBMDMs).

#### Experimental animals

All animal experiments were performed under UK Home Office licensing. For in vivo experiments males and females of at least 6 weeks of age were used, and experimental groups were age-matched. All mice were maintained in SPF conditions in IVC cages at the University of Cambridge. Mice were maintained on a 12-hour light/dark cycle; food and water were available ad libitum. Genotyping was by standard PCR protocols. Mice were bred and maintained in groups of 1-5 animals per cage; for skin wounding experiments, mice were singly housed. IL-1αTM mice were generated by homologous recombination of the point mutation into exon 5 of *Il1a* in a C57BL/6 FLP ES cell line using an FRT flanked Neo selection cassette, and generation of chimaeras with C57BL/6J mice (inGenious Targeting Laboratory). IL-1αTM mice were born at expected frequencies with no gross phenotype compared to littermate controls. IL-1R1^-/-^ mice were purchased from The Jackson Laboratory.

#### Human studies

Whole blood for plasma or serum, platelets, and monocytes was taken from healthy adult donors with informed consent and ethical approval from the National Research Ethics Service. Experiments used blood from both male and female donors with no age restrictions (See Table S2 and S3 for ARDS patient details and clinical parameters).

### Method Details

#### Cell-based assays

Necrotic lysates were made by freeze/thawing cells in liquid N_2_. Cells were treated with IL-1α (10 ng/ml; Peprotech); calpeptin (30 μM; Enzo); LPS (1 μg/ml); nigericin (15μM; Invivogen). IL-1α-specific activity was inferred by IL-6 production from HeLa or mouse fibroblasts in response to test treatments ±neutralising antibody against human (1μg/ml; Peprotech) or mouse (2μg/ml; R&D) IL-1α, or human IL-1β (1μg/ml; R&D). Conditioned media was collected, clarified, and IL-6 assayed as below.

#### In vivo experiments

Platelets were depleted by IV injection of CD42b (1.8μg/g; Emfret), with blood sampling from dorsal pedal veins. Thrombin was inhibited by oral gavage of dabigatran etexilate (100mg/kg). MKs were identified as FSC/SSC high, CD41^+^/61^+^ (both 1:200; Biolegend). For wound experiments, four full-thickness excisional wounds in shaved dorsal skin of isoflurane anaesthetised mice were made with a 4 mm biopsy punch (Integra). Mice had wounds photographed including a ruler for calibration, and wound area was determined with ImageJ. Healing rate constants were calculated by a one-phase decay model of curve fitting for wound area (GraphPad). Skin was dissected, formalin fixed (10%; 16h) and paraffin embedded. Wound exudate was absorbed onto filter paper disks, before soaking in PBS and separation by centrifugation. For endotoxemia, mice were injected IP with LPS (20 mg/kg; E. coli 0111:B4) and serum collected (+6 h). Endotoxemia was graded using a standard mouse monitoring sheet.

#### Mouse phenotyping

Blood counts utilised a Vet ABC counter (Scil), with clotting parameters analysed by ROTEM. Spleens were sieved (70μm) before washing (PBS; 350 g, 5 mins), re-sieving (40μm), RBC lysis (eBioscience), washing, and resuspension in FACs buffer (1% BSA, 0.05% NaN_3,_ in PBS) or full RPMI. Cells in FACs buffer were Fc blocked (1:100; Biolegend; 10 mins, RT) before staining for T cell activation with: anti-CD4 (1:800; eBioscience), anti-CD8 (1:100), anti-CD62L (1:80), anti-CD44 (1:400; all Biolegend), RT for 20 mins; or for Tregs with: anti-CD4 (1:800), anti-CD25 (1:80; Biolegend), RT for 20 mins, before washing, fixation, permeabilisation (FOXP3 Fix/Perm; Biolegend), then anti-Foxp3 (1:20; Biolegend), RT for 30 mins. Cells in RPMI were treated ±ionomycin/PMA/brefeldin A (1:500; Biolegend), incubated (5 h, 37°C), washed, resuspended in FACs buffer, Fc blocked, stained with anti-CD4 or anti-CD8 (20 mins, RT), washed, fixed, permeabilised (Biolegend), then anti-IL-10, anti-IL-17, and anti-IFNγ (all 1:100; Biolegend), RT for 30 mins. For neutrophil, monocyte or Ly6C^+^ cells, whole blood (EDTA) was stained with anti-CD115 (1:100; eBioscience), anti-CD11b (1:800), anti-Ly6G (1:80; both Biolegend), anti-Ly6C (1:400; abD Serotec), RT for 30 mins, before RBC lysis, washing, and analysis by flow cytometry (Accuri C6).

#### Platelet experiments

Platelets were obtained by centrifugation (280g, 20 min) of blood (sodium citrate; 0.38%) to give platelet-rich plasma (PRP). PRP was treated with p33, ± calpeptin (30μM), before addition of CaCl_2_ (20mM) and incubation (∼30 min, 37°C) to produce serum. PRP was washed by diluting 5 fold in HBSS (w/o Ca^2+^, pH 6.4, 4mM EDTA), centrifugation (280g, 20 min), before resuspension in HBSS. Platelets were treated with collagen-related peptide (50 μg/ml) or collagen (1 μg/ml; Helena), incubated (4-6 h, 37°C), stained with anti-hIL-1α-FITC (1:20; R&D), anti-hCD41-PE (0.63 μg/ml; Biolegend); or anti-mIL-1α-PE (1:20; Biolegend), anti-mCD41-FITC (1:200; Biolegend; 30 mins), before dilution (1:10; 1% BSA/PBS) and analysis by flow cytometry (Accuri C6), or thrombin treatment of supernatants or NP-40 (0.5%/PBS) lysates. Platelet were counted in whole blood (EDTA), stained with anti-CD41-FITC (1:200; Biolegend), diluted (1:1005; 1% BSA/PBS) and enumerated by flow cytometry.

#### Protein expression and purification

Human p33 (1-271), p17 (119-271), p18 (113-271) and mouse p33 (1-270), p17 (121-270) and p18 (115-270) was cloned into pGEX-4T-3 (GE) and mutations introduced by site directed mutagenesis. For GST purification, IPTG-induced cultures (Rosetta) were lysed in 50 mM Na_2_HPO_4_ pH 7.5, 150 mM NaCl, 1 mM DTT, 1 mM EDTA, with benzonase, lysozyme, and protease inhibitors (30 mins, RT), clarified, loaded onto a GSTrap column (GE) using an FPLC system (AKTA pure), washed, eluted (50 mM reduced glutathione), concentrated and dialyzed against 10 mM Tris pH 8.0, 50 mM NaCl. Protein concentration and purity was checked by Coomassie staining and quantified with an Odyssey scanner (Licor). Proteins were stored with 10% glycerol (-80°C).

#### Protease cleavage

p33 (20ng) was incubated in duplicate with calpain (50U; Calbiochem) in 10 mM Tris pH 7.5, 150 mM NaCl, 1 mM DTT, 2 mM CaCl_2_ (30 mins, RT); or restriction grade thrombin (0.09U, or as indicated; Merck) in 20 mM Tris pH 8.4, 150 mM NaCl, 2.5 mM CaCl_2_ (2 h, RT). For both, an enzyme control without p33, and an incubation control without enzyme was included. p33 cleavage was antagonised with a 100-fold molar excess of IL-1R2 (42 μg/ml; R&D), ± IL-1RAcP (50 μg/ml; Sino). p33 (150ng) was incubated with chymase (0.0003 U) in 27 mM Tris pH 7.7, 150 mM NaCl; elastase (0.03 U; Enzo) in 50 mM Tris pH 8.8 (both 2 h, RT); or granzyme B (16 μg/ml) in PBS (1 h, RT). Cell-derived p33 was extracted from LPS-treated (1μg/ml; 6 h, 37°C) mBMDMs or from keratinocyte conditioned media. Reactions were ±calpeptin (30μM) or ±PPACK (100μM; both Enzo).

#### Western blotting and Edman degradation

Westerns were performed as previously described with cells lysed directly in Laemmli buffer or RIPA (ThermoFisher) with protease inhibitors, followed by SDS-PAGE, transfer to PVDF membrane, blocking (5% milk), incubation (16h, 4°C) with human IL-1α pAb (1:500; Peprotech), mouse IL-1α (1:500; R&D) or IL-1β pAb (1:1000; Peprotech), or α/β-tubulin pAb (1:3333; CST), washing (PBS/Tween) and incubation (1 h, RT) with anti-rabbit HRP (1:2000; GE) or anti-goat HRP (1:2000; Jackson Immunoresearch). Visualisation was with ECL reagent (Amersham) and X-ray film (ThermoFisher). To analyse IL-1α/β in mBMDMs, calpeptin was added before lysis (30μM; 30 mins, 37°C) and to the lysis buffer (100μM). Thrombin cleaved IL-1α was resolved, transferred to PVDF, Coomassie stained, and ∼10 pmol of the C-terminal band sent for Edman degradation (Alta Bioscience).

#### Cytokine measurement and ELISA development

Samples were analysed by plate ELISA for human or mouse IL-1α (both Peprotech), cytometric bead ELISA for human or mouse IL-6 (BD), IL-1α or IL-1β (ThermoFisher), or mouse TAT complexes (Abcam). Specificity of human **(Figure S4I)** or mouse **(Figure S4J)** cleaved-IL-1α specific ELISA was proven with recombinant and/or cell-derived p17 and p33. The p18-specific antibody was produced in rabbits using SAPFSFLS-Ahx-KC-KLH for immunisation (Innovagen), and affinity purified. For indirect ELISA, plates (Maxisorb) were coated with peptide, p17, p18 or p33 (1 μg/ml; 16h, RT), washed (0.05% Tween/PBS), blocked (1% BSA; 1 h, RT), washed, serial diluted antiserum added (2 h, RT), washed, anti-rabbit-HRP antibody (1:5000; Dako) added (1 h, RT), washed and developed with TMB. For the p18-specific sandwich ELISA, plates (Costar) were coated with IL-1α capture antibody (R&D; 1μg/ml; 16h, RT), washed, blocked, samples added (2 h, RT), washed, biotinylated p18 detection antibody added (400 ng/ml; 2 h, RT), washed, anti-rabbit biotin added (1:7500; Dako; 1 h, RT), washed, streptavidin-HRP added (1:200; R&D; 1 h, RT), and developed with TMB (ThermoFisher). Plates were measured at 450nm (BioTek) and data analysed using a 4 parameter logistic standard curve. Human plasma was taken with informed consent and ethical approval, and cytokines measured (Mesoscale Discovery).

#### Cell and tissue staining

Paraffin embedded human (Abcam) or mouse skin sections were cleared, antigen retrieved with sodium citrate (10mM; pH 6), blocking in H_2_O_2_ (3%; 10 mins) and then horse serum (5%; 1 h), incubated with anti-IL-1α (1:100); anti-tissue factor (1:25; both Abcam); anti-Ly6G (1:500; Biolegend); anti-Mac-3 (1:400; BD); or isotype controls (Abcam; all 16 h, 4°C). Mac-3 and Ly6G sections were incubated with biotinylated 2ry antibodies (1:500; 1 h), ABComplex (30 mins), and visualised with DAB (all Vector), while IL-1α and tissue factor were visualised with Envision Kit (Dako) or a mouse/rabbit HRP/DAB kit (Abcam). Collagen was stained with picosirius red (Polysciences). Imaging used a BX51 (Olympus) and Image-Pro Insight 9.1 software (MediaCybernetics). Collagen fibre anisotropy was measured with ImageJ FibrilTool. Surface IL-1α on Accutase harvested, Fc blocked (1:100; Biolegend) human/mouse cells was stained with anti-hIL-1α-FITC (1:20; R&D), or anti-mIL-1α-PE (1:20; Biolegend) before flow cytometry.

### Quantification and Statistical Analysis

Data are presented as mean ± SEM, unless otherwise stated. Assays producing continuous data, with the exception of flow cytometry and in vivo mouse experiments, were in duplicate. n = individual experimental replicate. Parametric test analysis of continuous data used ANOVA or Student’s t-test (GraphPad). Non continuous data was analysed with Spearman’s rank correlation coefficient. Significance is as stated, but always p = <0.05. Non-significant (NS) data was considered anything p = >0.05. Datasets from the current study are available on reasonable request.

## Supplemental Information

Document S1. Figures S1–S4 and Tables S1–S3

Document S2. Article plus Supplemental Information
