# Supplementary material for: The Coagulation and Immune Systems Are Directly Linked through the Activation of Interleukin-1α by Thrombin
Source: Immunity. 2019 Apr 16;50(4):1033–1042.e6. doi: 10.1016/j.immuni.2019.03.003 (PMC6476404; doi:10.1016/j.immuni.2019.03.003)
Supplement: Document S1. Figures S1–S4 and Tables S1–S3 [file mmc1.pdf]

Immunity, Volume *50*

Supplemental Information

The Coagulation and Immune Systems Are Directly Linked through the Activation of Interleukin-1α by Thrombin

Laura C. Burzynski, Melanie Humphry, Katerina Pyrillou, Kimberley A. Wiggins, Julie N.E. Chan, Nichola Figg, Lauren L. Kitt, Charlotte Summers, Kate C. Tatham, Paul B. Martin, Martin R. Bennett, and Murray C.H. Clarke

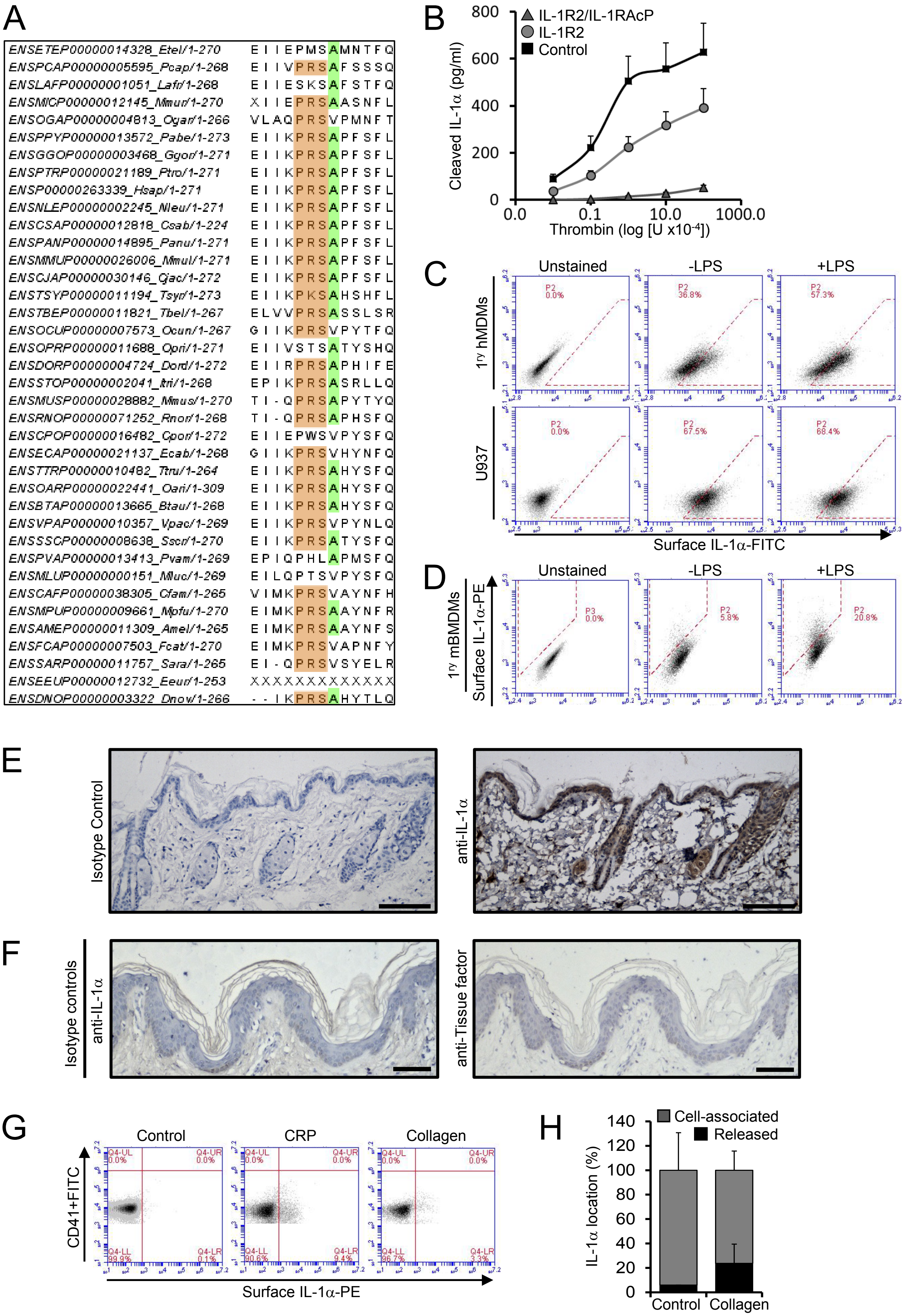
A
ENSETEP00000014328_Etel/1-270 EIIEPMSAMNTFQ
ENSPCAP00000005595_Pcap/1-268 EIIVPRSAFSSSQ
ENSLAFP00000001051_Lafr/1-268 EIIESKSAFSTFQ
ENSMICP00000012145_Mmur/1-270 XIIEPRSAASNFL
ENSOGAP00000004813_Ogar/1-266 VLAQPRSVPMNFT
ENSPPYP00000013572_Pabe/1-273 EIIKPRSAPFSFL
ENSGGOP00000003468_Ggor/1-271 EIIKPRSAPFSFL
ENSPTRP00000021189_Ptro/1-271 EIIKPRSAPFSFL
ENSP00000263339_Hsap/1-271 EIIKPRSAPFSFL
ENSNLEP00000002245_Nleu/1-271 EIIKPRSAPFSFL
ENSCSAP00000012818_Csab/1-224 EIIKPRSAPFSFL
ENSPANP00000014895_Panu/1-271 EIIKPRSAPFSFL
ENSMMUP00000026006_Mmul/1-271 EIIKPRSAPFSFL
ENSCJAP00000030146_Cjac/1-272 EIIKPRSAPFSFL
ENSTSYP00000011194_Tsyr/1-273 EIIKPKSAHSHFL
ENSTBEP00000011821_Tbel/1-267 ELVVPRSASSLSR
ENSOCUP00000007573_Ocun/1-267 GIIKPRSVPYTFQ
ENSOPRP00000011688_Opri/1-271 EIIVSTSATYSHQ
ENSDORP00000004724_Dord/1-272 EIIRPRSAPHIFE
ENSSTOP00000002041_Itri/1-268 EPIKPRSASRLLQ
ENSMUSP00000028882_Mmus/1-270 TI-QPRSAPYTYQ
ENSRNOP00000071252_Rnor/1-268 TI-QPRSAPHSFQ
ENSCPOP00000016482_Cpor/1-272 EIIEPWSVPYSFQ
ENSECAP00000021137_Ecab/1-268 GIIKPRSVHYNFQ
ENSTTRP00000010482_Ttru/1-264 EIIKPRSAHYSFQ
ENSOARP00000022441_Oari/1-309 EIIKPRSAHYSFQ
ENSBTAP00000013665_Btau/1-268 EIIKPRSAHYSFQ
ENSVPAP00000010357_Vpac/1-269 EIIKPRSVPYNLQ
ENSSSCP00000008638_Sscr/1-270 EIIKPRSATYSFQ
ENSPVAP00000013413_Pvam/1-269 EPIQPHLAPMSFQ
ENSMLUP00000000151_Mluc/1-269 EILQPTSVPYSFQ
ENSCAFP00000038305_Cfam/1-265 VIMKPRSVAYNFH
ENSMPUP00000009661_Mpfu/1-270 EIMKPRSAAYNFR
ENSAMEP00000011309_Amel/1-265 EIMKPRSAAYNFS
ENSFCAP00000007503_Fcat/1-270 EIMKPRSVAPNFY
ENSSARP00000011757_Sara/1-265 EI-QPRSVSYELR
ENSEEUP00000012732_Eeur/1-253 XXXXXXXXXXXXX
ENSDNOP00000003322_Dnov/1-266 --IKPRSAHYTLQ
B
IL-1R2/IL-1RAcP
IL-1R2
Control
Cleaved IL-1α (pg/ml)
800
600
400
200
0
0.0
0.1
10.0
1000.0
Thrombin (log [U x10⁻⁴])
C
Unstained
-LPS
+LPS
1ry hMDMs
U937
P2 0.0%
P2 36.8%
P2 57.3%
P2 0.0%
P2 67.5%
P2 68.4%
Surface IL-1α-FITC
D
Unstained
-LPS
+LPS
1ry mBMDMs
Surface IL-1α-PE
P3 0.0%
P2 5.8%
P2 20.8%
E
Isotype Control
anti-IL-1α
F
Isotype controls
anti-IL-1α
anti-Tissue factor
G
Control
CRP
Collagen
CD41+FITC
Q4-UL 0.0%
Q4-UR 0.0%
Q4-LL 99.9%
Q4-LR 0.1%
Q4-UL 0.0%
Q4-UR 0.0%
Q4-LL 90.6%
Q4-LR 9.4%
Q4-UL 0.0%
Q4-UR 0.0%
Q4-LL 96.7%
Q4-LR 3.3%
Surface IL-1α-PE
H
Cell-associated
Released
IL-1α location (%)
140
120
100
80
60
40
20
0
Control
Collagen

Supplemental Figure S1, related to Figure 1 and 2: (A) Protein alignment of all available IL-1α sequences showing conservation of a PRS motif in ~83% of mammals. **(B)** Cleaved IL-1α-specific ELISA showing reduced thrombin cleavage of IL-1α by IL-1R2 or IL-1R2/IL-1RAcP at a 100-fold molar excess. **(C,D)** Flow cytometry for surface IL-1α in primary human macrophages (hMDMs) and monocytic U937 cells **(C)**, or primary murine macrophages (mBMDMs) **(D)**, treated ± LPS. **(E)** Mouse skin stained with anti-IL-1α (brown) or isotype control antibodies. **(F)** Human skin stained with isotype control antibodies for anti-IL-1α or anti-tissue factor (both brown). **(G)** Flow cytometry for CD41 and IL-1α on resting and collagen or collagen-related peptide (CRP) treated murine platelets. **(H)** Cleaved IL-1α-specific ELISA showing increased IL-1α release from the surface of activated human platelets by thrombin. Data represent mean ± SEM; n = 2 **(H)**. Scale bars represent 100 μm.

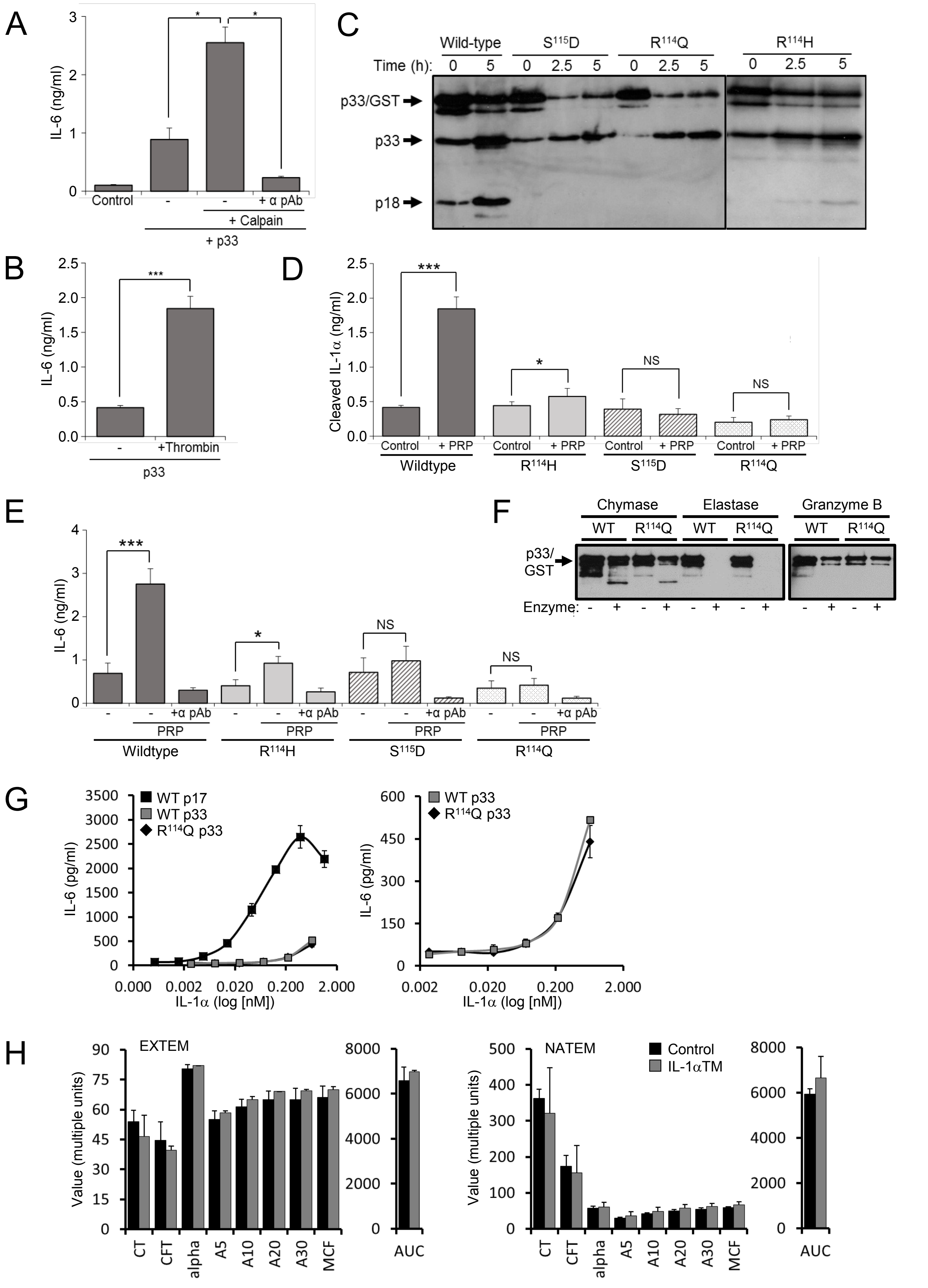
A
B
C
D
E
F
G
H
IL-6 (ng/ml)
Control
+ α pAb
+ Calpain
+ p33
+Thrombin
p33
Wild-type
S115D
R114Q
R114H
Time (h):
p33/GST
p33
p18
Cleaved IL-1α (ng/ml)
Control
+ PRP
Wildtype
NS
PRP
Chymase
Elastase
Granzyme B
WT
Enzyme
WT p17
WT p33
R114Q p33
IL-6 (pg/ml)
IL-1α (log [nM])
EXTEM
NATEM
Control
IL-1αTM
Value (multiple units)
CT
CFT
alpha
A5
A10
A20
A30
MCF
AUC

Supplemental Figure S2, related to Figure 3: (A,B) IL-1-dependent IL-6 production by murine fibroblasts incubated with calpain **(A)** or thrombin **(B)** cleaved mouse p33 IL-1α, ± a neutralising IL-1α pAb (+α pAb). **(C)** Western blot for IL-1α showing thrombin cleavage of wild type (WT) or mutant forms of mouse p33. **(D,E)** Cleavage and activation of WT or mutant forms of mouse p33 during clotting of platelet-rich plasma (PRP). **(F)** Western blot for IL-1α showing equivalent processing of mouse WT or $R^{114}Q$ mutant p33 by proteases. **(G)** IL-1-dependent IL-6 production by murine fibroblasts incubated with increasing concentrations of recombinant murine WT p17, WT p33, or $R^{114}Q$ p33. **(H)** Rotational thromboelastometry (ROTEM) analysis of clotting in whole blood from control or IL-1αTM mice. CT = clotting time (s); CFT = clot formation time (s); alpha = alpha angle (˚); A5 - A30 = amplitude at 5 – 30 mins; MCF = maximal clot firmness; AUC = area under the curve; EXTEM = extrinsic TEM (tissue factor stimulation); NATEM = non-activated TEM. Data represent mean ± SEM, n = 3 **(A,D,E)**, n = 6 **(B)**; mean ± SD representative of n = 2 **(G)**; or mean ± SD, n = 2 **(H)**; p = *≤0.05, ***≤0.001; NS = not significant.

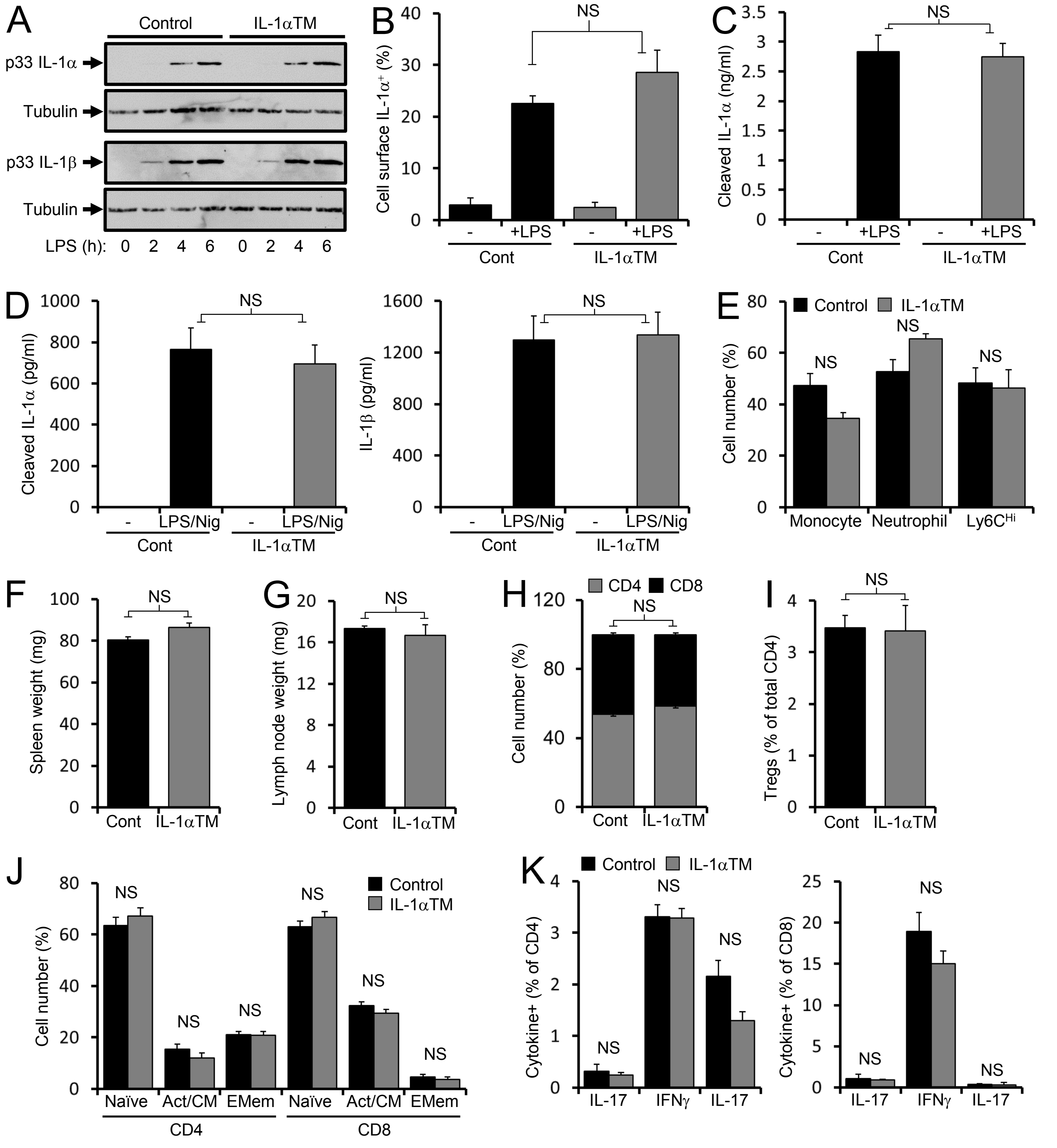

Supplemental Figure S3, related to Figure 3: (A-C) Macrophages (BMDMs) from control and IL-1αTM mice were treated ±LPS and analysed by western blot for IL-1α/β expression **(A)**, by flow cytometry for cell surface IL-1α **(B)**, and by ELISA for cleaved IL-1α after necrosis **(C)**. **(D)** ELISA data for IL-1α/β release from mBMDMs after inflammasome activation with LPS followed by Nigericin (LPS/Nig). **(E)** Flow cytometry for blood neutrophils, monocytes and Ly6C^{Hi} monocytes in mice. **(F-K)** Analysis for spleen **(F)** and lymph node weight **(G)**, percentage of CD4/8 T cells **(H)** and Tregs **(I)** in the spleen, splenic CD4/8 T cell activation status **(J)**, and splenic CD4/8 T cell cytokine expression after PMA/ionomycin activation **(K)**. Act/CM = activated/conditional memory; Emem = effector memory. Data represent mean ± SEM, n = 3 **(B,C,E-K)**, n = ≥5 **(D)**; NS = not significant.

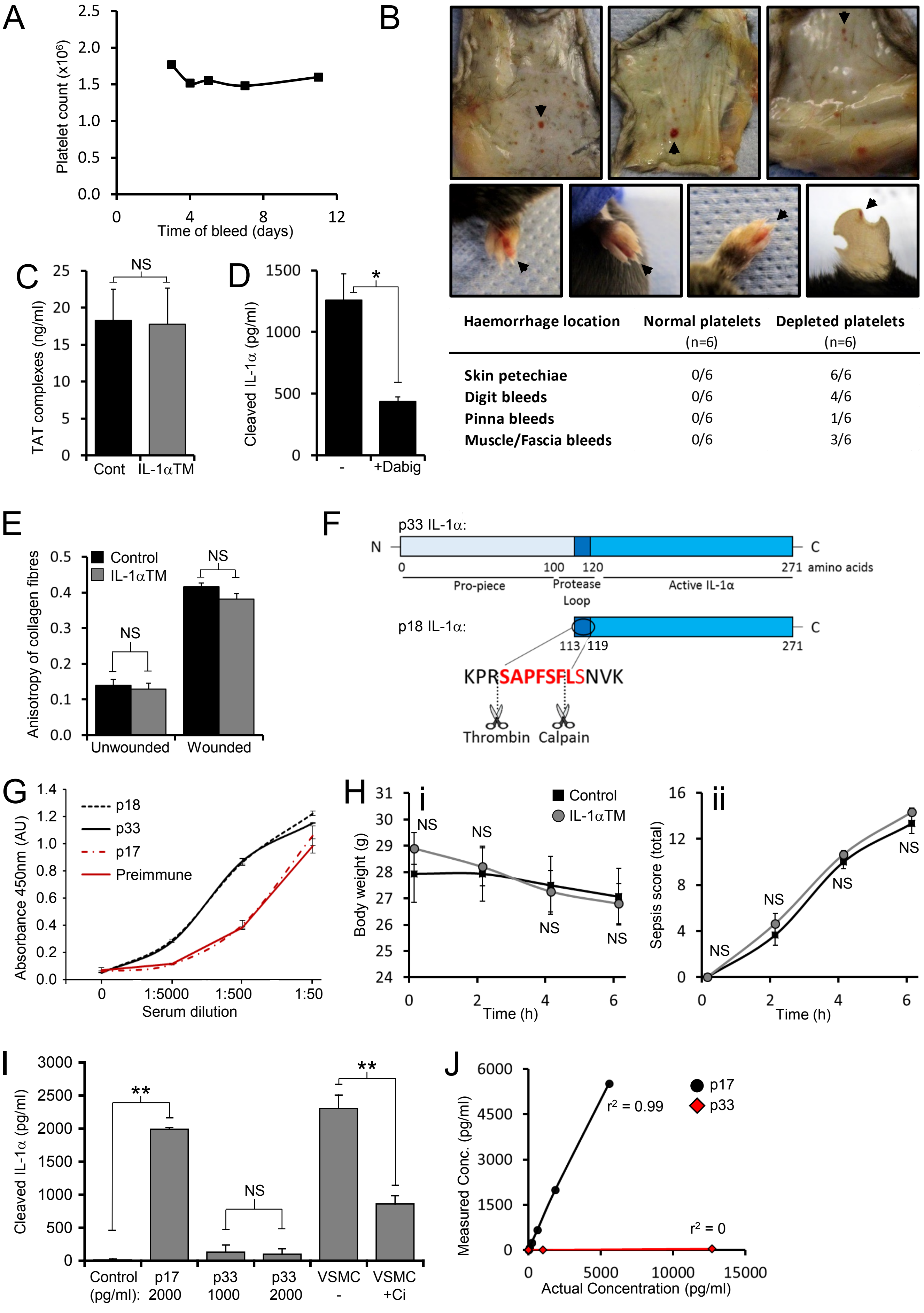

Haemorrhage location	Normal platelets (n=6)	Depleted platelets (n=6)
**Skin petechiae**	0/6	6/6
**Digit bleeds**	0/6	4/6
**Pinna bleeds**	0/6	1/6
**Muscle/Fascia bleeds**	0/6	3/6

Supplemental Figure S4, related to Figure 4 and STAR Methods: (A) Platelet count over time in control mice without platelet depletion. **(B)** Representative images showing multiple haemorrhages in the skin (petechiae), digits and ear pinna (arrows) of control mice 24 h after platelet depletion, along with bleed tally. **(C)** ELISA data for thrombin-antithrombin (TAT) complexes in wound exudate from control and IL-1αTM mice. **(D)** ELISA data for level of cleaved IL-1α in wound exudate from control mice treated ± dabigatran (Dabig). **(E)** Scarring 14 d after excisional skin wounding measured by parallel collagen fibre alignment. **(F)** Schematic showing the calpain and thrombin cleavage sites and the inoculating peptide sequence (red) for p18 antibody production. **(G)** Indirect ELISA data showing reactivity of anti-p18 antiserum for p17, p18 or p33 IL-1α, and the signal from preimmune serum only. **(H)** Body weight **(i)** and sepsis severity scores over time for mice injected intraperitoneally with LPS and assessed undisturbed or handled to give a total sepsis severity score **(ii). (I)** Specificity of the cleaved IL-1α-specific ELISA for detection of recombinant human p17 and p33, and VSMC-derived p17 and p33 IL-1α by using a calpain inhibitor (+Ci) to prevent p17 generation. **(J)** Data showing specificity of the cleaved IL-1α-specific ELISA for detection of recombinant murine p17 and p33 IL-1α. Data represent mean ± SEM; n = 2 **(I)**, n = 3 **(C,H,J)**, n = ≥5 **(D)**, n = ≥21 wounds **(E)**, n = 2 **(G)**; p = *≤0.05, p = **≤0.01; NS = not significant.

	Control (n=7)	IL-1αTM (n=16)	p =
**WBC (10^3/mm^3)**	7.03 ± 1.03	7.64 ± 0.41	0.60
**RBC (10^6/mm^3)**	10.01 ± 0.71	11.42 ± 0.32	0.10
**Haemoglobin (g/dL)**	13.21 ± 1.02	14.05 ± 0.76	0.52
**Haematocrit (%)**	56.96 ± 4.40	62.82 ± 1.78	0.25
**MCV (fm^3)**	56.71 ± 1.06	55.63 ± 0.44	0.37
**Platelets (10^3/mm^3)**	1153.14 ± 114.67	1104.13 ± 92.01	0.74
**MPV (fm^3)**	5.07 ± 0.12	4.80 ± 0.09	0.09
**Lymphocytes (%)**	83.43 ± 1.66	85.68 ± 0.98	0.27
**Granulocytes (%)**	12.37 ± 1.22	10.31 ± 0.81	0.18
**Monocytes (%)**	4.20 ± 0.47	4.01 ± 0.25	0.72

Supplemental Table S1. Mouse full blood counts, related to Figure 3: Full blood counts using an autoanalyser in whole blood from control or IL-1αTM mice. MCV = mean corpuscular volume; MPV = mean platelet volume. Data represent mean ± SEM.

	ARDS (n=9)	Control (n=9)	Fold change	p =
**Age**	47.1 ± 6.8	49 ± 8.2	-	0.47
**Sex - Female (%)**	33	56	-	0.37
**SAA (μg/ml)**	207.4 ± 144.6	19.9 ± 55.6	10.4	**0.001**
**CRP (μg/ml)**	172.1 ± 46.9	9.2 ± 25.0	18.6	**0.00000003**
**IL-6 (pg/ml)**	60.0 ± 89.3	1.0 ± 0.9	61.9	**0.05**
**MCP-1 (pg/ml)**	596.6 ± 599.1	144.8 ± 22.3	4.1	**0.03**
**VCAM-1 (μg/ml)**	1.7 ± 1.5	0.5 ± 0.5	3.4	**0.02**
		(reference range)		
**Haemoglobin (g/L)**	108.9 ± 24.9	130-180	~0.70	-
**Haematocrit (%)**	0.32 ± 0.08	0.40-0.52	~0.69	-
**Platelet count (10^9/L)**	152.4 ± 105.4	150-450	~0.51	-
**Plateletcrit (%)**	0.15 ± 0.10	0.22-0.44	~0.45	-
**APTT (s)**	41.3 ± 14.0	23-30	~1.56	-
**Prothrombin time (s)**	20.5 ± 9.6	10-13	~1.78	-

Supplemental Table S2. Clinical characteristics of sepsis-induced ARDS patients and control individuals, related to Figure 4: Electrochemiluminescence immunoassay for level of soluble inflammatory mediators in serum from control individuals or ARDS patients, along with clinical haematology parameters for ARDS patients and normal reference range (95% confidence intervals). SAA = serum amyloid A; CRP = c-reactive protein; MCP-1 = monocyte chemoattractant protein 1; VCAM-1 = vascular cell adhesion molecule 1; APTT = activated partial thromboplastin time. Data represent mean ± SD.

	ARDS p18+ (n=5)	ARDS p18- (n=4)	p =
**Age**	43.0 ± 6.6	52.3 ± 1.3	**0.03**
**Sex - Female (%)**	20	50	NS
**Weight (kg)**	78.9 ± 13.2	71.8 ± 7.1	NS
**Height (cm)**	171.3 ± 13.2	159.7 ± 9.0	NS
**WBC ($10^3/mm^3$)**	13.0 ± 5.3	13.9 ± 12.4	NS
**PMN ($10^3/mm^3$)**	10.7 ± 5.0	13.0 ± 11.9	NS
**PEEP (cmH_2O)**	9.0 ± 1.4	6.0 ± 1.4	**0.016**
**Microbiol. +VE (%)**	5/5	1/4	**0.01**

Supplemental Table S3. Clinical characteristics of sepsis-induced ARDS patients divided between those positive and negative for p18 IL-1α, related to Figure 4: WBC = white blood cell; PEEP = positive end-expiratory pressure; Microbiol. +ve = +ve microbiology in lung bronchoalveolar lavage fluid. Data represent mean ± SD.
